# SufB intein splicing in *Mycobacterium tuberculosis* is influenced by two remote conserved N-extein histidines

**DOI:** 10.1042/BSR20212207

**Published:** 2022-03-02

**Authors:** Sunita Panda, Ananya Nanda, Nilanjan Sahu, Deepak K. Ojha, Biswaranjan Pradhan, Anjali Rai, Amol R. Suryawanshi, Nilesh Banavali, Sasmita Nayak

**Affiliations:** 1 School of Biotechnology, Kalinga Institute of Industrial Technology, Bhubaneswar 751024, Odisha, India; 2 School of Biological Sciences, National Institute of Science Education and Research Bhubaneswar, Jatni, Khurda 752050, Odisha, India; 3 S.K. Dash Center of Excellence of Biosciences and Engineering and Technology, School of Basic Sciences, Indian Institute of Technology Bhubaneswar, Argul, Khurda 752050, Odisha, India; 4 Clinical Proteomics, Institute of Life Sciences, Bhubaneswar 751023, Odisha, India; 5 Laboratory of Cellular and Molecular Basis of Diseases, Division of Transitional Medicine, Wadsworth Center, New York State Department of Health, Empire State Plaza, Albany 12237, NY, U.S.A.; 6 Department of Biomedical Sciences, School of Public Health, 1 University Place, Rensselaer 12144, NY, U.S.A.

**Keywords:** Conserved histidines, Intein splicing, Mycobacterium tuberculosis SufB, Splicing regulation, SufB intein

## Abstract

Inteins are auto-processing domains that implement a multistep biochemical reaction termed protein splicing, marked by cleavage and formation of peptide bonds. They excise from a precursor protein, generating a functional protein via covalent bonding of flanking exteins. We report the kinetic study of splicing and cleavage reaction in [Fe–S] cluster assembly protein SufB from *Mycobacterium tuberculosis* (*Mtu*). Although it follows a canonical intein splicing pathway, distinct features are added by extein residues present in the active site. Sequence analysis identified two conserved histidines in the N-extein region; His-5 and His-38. Kinetic analyses of His-5Ala and His-38Ala SufB mutants exhibited significant reductions in splicing and cleavage rates relative to the SufB wildtype (WT) precursor protein. Structural analysis and molecular dynamics (MD) simulations suggested that *Mtu* SufB displays a unique mechanism where two remote histidines work concurrently to facilitate N-terminal cleavage reaction. His-38 is stabilized by the solvent-exposed His-5, and can impact N–S acyl shift by direct interaction with the catalytic Cys1. Development of inteins as biotechnological tools or as pathogen-specific novel antimicrobial targets requires a more complete understanding of such unexpected roles of conserved extein residues in protein splicing.

## Introduction

**Figure 1:**
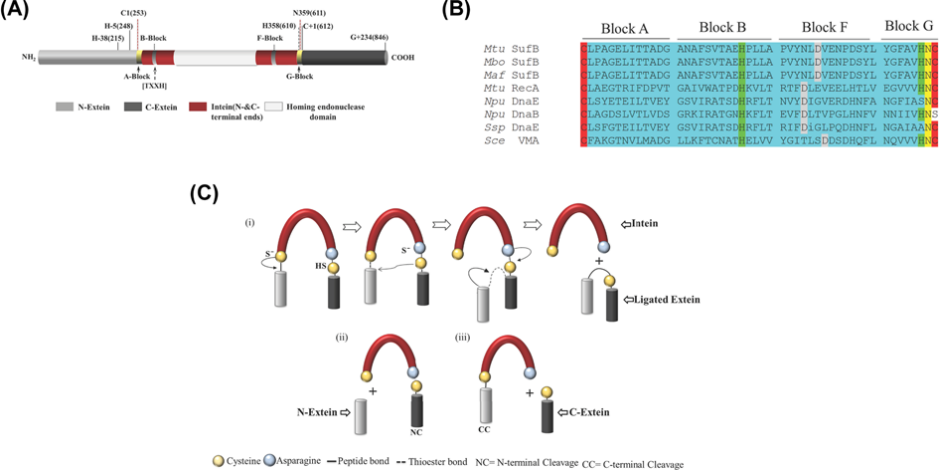
Conservation of residues within the structural domains of *Mtu* full-length (FL) SufB precursor (**A**) Schematic representation of different structural domains of the *Mtu* FL-SufB precursor. Important residues for the intein (numbered 1, 2, …), N-extein (numbered −1, −2, …) and C-extein (numbered +1, +2, …) are shown at the top. The conserved N-extein histidines are H-5 (H248 in *Mtu* FL-SufB) and H-38 (H215 in *Mtu* Fl-SufB). (**B**) Multiple sequence alignment of *Mtu* SufB with other intein-containing precursors shows conserved residues (highlighted in different colors) that participate in a canonical splicing pathway. (**C**) (i) Mechanism of intein splicing comprising sequential nucleophilic attacks by C1 and C+1 to generate a branched intermediate by transesterification, cyclization of terminal Asn to resolve the branched intermediate ligates exteins and to cleave the SufB intein. (ii) N-terminal and (iii) C-terminal cleavage to form off-pathway products.

Protein splicing is a self-catalyzed event that generates a continuous extein protein by ligating two intein separated extein regions with a peptide bond. This post-translational auto-excision of the intervening intein protein is critical for the formation of an active protein [1–4]. Splicing and cleavage products (Figure 1C) are obtained through a series of nucleophilic displacement reactions mediated in a coordinated fashion by the catalytic residues [5–9]. Control over this reversible interruption of the functional form of the host exteins can play a regulatory role in protein activation [10,11]. The extein sequences upstream and downstream of N-terminal and C-terminal intein ends are termed ‘N-extein’ and ‘C-extein’ respectively. The intein folds into a horseshoe-shaped structure with a catalytic cleft that brings the conserved catalytic residues and the extein splice junction close enough for initiation of splicing reaction [12].

Inteins themselves have N-terminal and C-terminal regions with conserved sequence segments (termed Blocks or motifs) that facilitate the splicing and cleavage reaction(s). N-terminal intein region comprises A, N2, B, and N4 structural motifs or Blocks while F- and G-Blocks are part of the C-terminal intein region [13]. Typically, A-Block contains Cys/Ser or Thr; B-Block includes His and Thr residues; F-Block usually has Asp and His and the G-Block bears two conserved residues; a penultimate His and a terminal Asn [8,14,15]. Classical or Canonical (Class 1) intein splicing involves four sequential acyl rearrangements (Figure 1C), a nucleophilic attack by C1 or S1/T1 leads to an N–S/N–O acyl shift converting the peptide bond of N-terminal splice junction into a thioester linkage, a second nucleophilic attack by C+1 forms a branched intermediate at C-terminal splice junction through esterification, the branched intermediate is resolved by terminal Asn cyclization through cleavage of C-terminal splice junction, and finally an (S–N/O–N) acyl shift fully ligates the two extein segments by an amide bond formation [16–18].

These bond rearrangements at splice junctions during the cleavage and splicing reaction(s) are assisted by noncatalytic intein residues through stabilization of various intermediate structure(s) [19]. The Block-A residues Cys, Ser, or Thr participate in the first step of splicing with significant assistance from the Block-B His and Thr residues. The highly conserved Block-B His destabilizes the scissile peptide bond either by reducing the energy barrier or by loss of resonance or protonation of the Cys1 amide bond via His imidazole ring to catalyze the N–S acyl shift [20–24]. The Block-F Asp residue drives the thioesterification through the tetrahedral intermediate by ground-state destabilization [8,22,25]. It is also proposed that Block-B histidine plays a dual catalytic role; being weakly basic it deprotonates the Cys1 to accelerate the N–S acyl shift and subsequently acts as an acid to stabilize the tetrahedral intermediate [21,23,24]. The Block-F Asp residue also deprotonates the C+1 residue to stabilizes the net positive charge on Cys1 that drives the transesterification reaction. The F- and G-Block His residues are critical in the coordination of terminal Asn cyclization. The F-Block His increases nucleophilicity of Asn, and the G-Block His accelerates the Asn cyclization by increasing the electrophilicity of backbone peptide [26–29]. The final acyl-shift is energetically favorable and does not require assistance from either intein or extein residues [30]. Inteins do show polymorphisms in the catalytic residues leading to variation in the splicing mechanism as seen in Class 2 and Class 3 intein splicing [11,31–35].

Interactions between extein residues and the catalytic intein core in the regulation of splicing reaction have been studied by modulating intein activity, changing the conformation of catalytic cleft, and restraining the activity of catalytic residues [6,15,36–39]. Previous studies on intein–extein partnership in intein splicing have suggested mediation by extein residues both near and remote to the N- and C-terminal splice junctions [7,15,36,38,40–42]. The N-extein residue at the first position (−1) is important for the first thioester reaction and shows enhanced N-terminal cleavage rate (more than four-fold) or attenuated cleavage by 1000-fold by replacing the native residue to aspartate and proline, respectively [36]. Substitution of bulky amino acids at this position can cause local distortion and induce N-cleavage reaction [23]. The participation of the first C-extein Cys+1 in the second and third steps of the splicing reaction has been demonstrated by its mutation dramatically augmenting or inhibiting splicing and generating off-pathway N-cleavage products [12,14,17,40,43]. Earlier, extein effects were assumed to be limited to residues proximal to the intein [12,15,44], but recent work has shown that distal exteins are implicated as environmental sensors with the role in regulating splicing depending on solution environment and temperature in *Pho* RadA precursors [6,10,39].

SufB is a critical component of [Fe–S] cluster assembly and repair machinery called SUF (mobilization of sulfur) complex. This is a stress response system that gets up-regulated during periods of oxidative stress and Fe starvation [45,46]. Though there are multiple pathways for [Fe–S] cluster biogenesis among the three kingdoms of life such as nitrogen fixation (NIF) and Iron–sulfur cluster (ISC), the SUF complex is unique in mycobacteria. [Fe–S] cluster containing proteins execute a broad spectrum of cellular functions in organisms such as respiration, gene regulation, RNA modification, DNA repair, and replication [47–49]. Although the SUF system has been well characterized in the *Escherichia coli* system [17,45,46,50–52], the importance of SufB intein splicing in the formation of functional Suf complex has been shown in mycobacteria [52].

**Figure 2:**
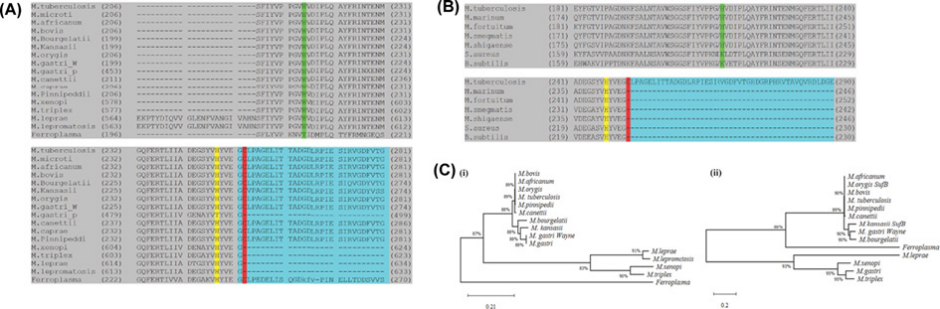
Conserved histidines in the *Mtu* FL-SufB N-extein (**A**) Multiple sequence alignment focused on the junction between the N-extein and intein in mycobacterial and archaeal species SufB proteins with conserved H-5 (yellow), H-38 (green), C1 in intein (red). N-extein and intein residues are delineated by shading in gray and cyan blue, (**B**) multiple sequence alignment focusing on N-extein–intein junction in mycobacteria and organisms where SUF is the exclusive system for [Fe–S] cluster assembly, (**C**) phylogenetic analyses of (i) full-length (FL) SufB protein and (ii) SufB intein sequences in mycobacteria and *Ferroplasma acidarmans*.

The present study reports on the splicing and cleavage reactions of a full-length *Mycobacterium tuberculosis* (*Mtu*) SufB (FL-SufB) precursor protein. We delineated the different structural domains of *Mtu* SufB, analyzed whether it follows a canonical or noncanonical intein splicing pathway, identified intein and extein residues that participate in catalytic cleft formation, assessed both their conservation in different mycobacterial species and their role in regulating cleavage and/or splicing reactions, and analyzed distinctions from other intein precursor proteins. We found intein residues highly conserved in different mycobacterial species that favor a canonical splicing mechanism (Figure 1A,B). We detected two distal histidines in the N-extein region, His-5 and His-38, that are conserved in mycobacteria, archaea, and other microbes where SUF is the exclusive system for [Fe–S] cluster biogenesis (Figure 2A,B). Biochemical analyses of H-5A and H-38A SufB mutants confirmed their influence on splicing and cleavage. Structural modeling of *Mtu* SufB and explicit-solvent molecular dynamics (MD) simulations of the model were used to analyze the SufB precursor splicing active site dynamics, and these simulations suggested that N-terminal cleavage could be supported by an interaction between H-38 and H-5. These observations suggest that the two distal H-5 and H-38 N-extein residues participate in SufB precursor stabilization and aid *Mtu* SufB intein splicing.

Kinetic analyses of H-5A SufB mutant demonstrated 3- and 1.4-fold reductions in splicing and cleavage rates, respectively, relative to wild-type *Mtu* SufB precursor. Likewise, 3.4- and 3-fold reduction in splicing and cleavage rates were observed in the H-38A SufB mutant. A side-by-side *Mtu* SufB structure prediction was done using homology (chimera) modeling, secondary structure prediction through consensus with other protein sequences. Subsequently, MD simulations in aqueous media were carried out to find the equilibrated structure. Furthermore, MD simulations clarified the structural features of the SufB intein active site and indicated that N-terminal cleavage reaction is catalyzed by H-38 with the assistance of H-5. Taken together, our study substantiates a distinct mechanism for N-terminal cleavage reaction shown by *Mtu* SufB. Although H-38 is relatively distal to the N-terminal splice junction when supported by His-5, it can efficiently activate the first step of splicing reaction. Finally, we have proposed a novel mechanism for the N-cleavage reaction mediated via the concerted actions of these conserved histidines in the N-extein region of *Mtu* FL-SufB precursor. These observations suggest that H-5 and H-38 might have important biological role(s) in the SufB precursor stabilization and perhaps the functionality of *Mtu* SufB protein.

## Materials and methods

### Genetic constructs

The full-length [Fe–S] cluster assembly protein SufB from the *Mycobacterium tuberculosis* H37Rv strain (*Mtu* FL-SufB) and its isolated intein (*Mtu*-SufB-I) genes were PCR-amplified using Pfu Ultra High-Fidelity DNA Polymerase (Agilent Technologies) from heat-killed *Mtu* genomic DNA. DNA purification by gel electrophoresis was followed by EcoRI and HindIII restriction digestion and cohesive end ligation (T4 DNA Ligase, NEB Cat. No. M0202S) for cloning. The genes were inserted into the multiple cloning site 1 (MCS1) of the low copy expression vector pACYCDuet™-1 (Novagen), which was driven by a T7 promoter/lac operator with a chloramphenicol resistance gene for selection. The constructs were screened via colony PCR and confirmed by sequencing (Sequencing Core Facility, SUNY, Albany, and Eaton Bioscience Inc. sequencing service) using the ACYCDuetUP1 (Novagen Cat. No. 71178-3) and DuetDOWN1 primers (Novagen Cat. No. 71179-3), as well as the original primers. *Mtu* FL-SufB mutants H-5A, H-38A, C1A, N359A were generated by substituting respective key catalytic residues to alanine via phosphorylated inverse PCR primers. Splicing inactive (SI) *Mtu* FL-SufB double mutant (C1A/N359A) was created via inverse PCR to add the N359A mutation into the C1A cleavage mutant. The cloned H-38A SufB mutant genes were also confirmed separately by sequencing (Agrogenomics, Odisha) using ACYCDuetUP1 (IDT Cat. No. 103948189) and DuetDOWN1 (IDT Cat. No. 103948190) along with the SufB primers. All the above primers are listed in Supplementary Table S1.

### Sequence analysis

Protein sequences for *Mtu* FL-SufB (Accession number YP_006514844.1, GI: 397673309) and the 477-amino acid intein-less SufB protein from *Mycobacterium smegmatis* strain MC2 155 (*Msm*-SufB-FL, accession number YP_887437.1, GI: 118472504) were pairwise sequence-aligned in ClustalW to distinguish extein and intein regions in *Mtu* FL*-*SufB [53]. The identified *Mtu* SufB intein sequence (*Mtu* SufB-I) was confirmed by sequence comparison using Blast with the sequences deposited in Inbase (The Intein Database, www.inteins.com) [54]. Different structural domains of the intein-like homing endonuclease, and the N- and C-terminal inteins were clearly demarcated by sequence alignment and structural analysis of SufB intein with homing endonuclease domain (I-CreI) and intein homing endonuclease Ii (PDB: 2CW7). The *Mtu* SufB-FL and *Msm*-SufB-FL sequences, combined with one archaeal and other bacterial SufB proteins collected from the NCBI and intein databases [54], were aligned using Dialign2 software [55]. Conservation of different intein and extein residues was edited and color coded manually. Phylogenetic tree analysis was performed using the Maximum likelihood method in the MEGA X program [56,57] for both SufB inteins [Figure 2C(ii)] and SufB precursor sequences [Figure 2C(i)] from different organisms.

### Protein overexpression and purification

Full-length (FL) unspliced precursor and mutant SufB proteins carrying an N-terminal 6×His-tag were overexpressed in BL21 (DE3) *E. coli* cells via isopropyl β-d-1-thiogalactopyranoside (IPTG; 500 µM) (Sigma 367-93-1) induction at 37°C for 4 h. Cells were resuspended in lysis buffer (20 mM sodium phosphate, 0.5 M NaCl, pH 7.4) and lysed via tip sonicator (Sonics vibra cell VCX-130). Proteins were overexpressed and isolated from inclusion bodies (IBs) via centrifugation. The IB materials were solubilized by 8 M urea [23] (Merck, 1084870500) buffer (lysis buffer, 8 M urea, 20 mM of imidazole (MP–biochemicals-288-32-4) and centrifuged at 16500×***g*** for 20 min to collect the supernatant. Then 6×His-tagged wildtype (WT) SufB precursor and mutant proteins were purified by Ni-NTA affinity column (Ni-NTA His trap, HP GE Healthcare Life Sciences-17524802) [23,58–62]. Prior to sample application, columns were equilibrated with binding buffer (20 mM sodium phosphate, 0.5 M NaCl, 40 mM imidazole). After sample loading, columns were washed several times (15 CV) in binding buffer. Finally, proteins were eluted as purified fractions in elution buffer (20 mM sodium phosphate, 0.5 M NaCl, 500 mM imidazole) followed by quantification via Bradford’s assay.

### *In vitro* splicing and cleavage assays

A total of 2.5 µM of purified proteins were allowed to refold in 1 ml of renaturation buffer (20 mM sodium phosphate, 0.5 M NaCl, 0.5 M arginine,1 mM EDTA, pH 7.4) in presence of 2 mM TCEP-HCl (Sigma-51805-45-9) at 20°C for 24 h [58–63]. The 0-h sample was retrieved before renaturation and splicing was quenched by addition of loading dye (0.1% Bromophenol Blue, 50% glycerol, β-mercaptoethanol, 10% sodium dodecyl sulfate (SDS), tris 6.8) followed by rapid freezing at −20°C. Our controls, SI SufB double mutant and empty expression vector pACYC Duet-1, were treated similarly for the *in vitro* assays. For the N-cleavage assay, proteins were refolded in presence of reducing agents and nucleophiles such as 2 mM TCEP-HCl, 50 mM dithiothreitol (DTT) (Roche-10708984001), and 0.5 M 250 Hydroxylamine (SRL-66164) with 1 mM Tris (2-carboxyethyl) phosphine (TCEP) in renaturation buffer [58–63]. For splicing and cleavage analysis, sample extraction at each time interval was followed by the addition of loading dye to stop the reactions and then boiling at 95°C for 5 min. Resultant products from various refolding reactions were resolved through 4–10% gradient SDS/polyacrylamide gel electrophoresis (PAGE). Protein bands were stained with Coomassie Blue R-250 and densitometric analysis was performed by using GelQuant.Net biochemical solutions. Percentage(s) of splicing and cleavage products were measured by taking the percentage(s) of the ratio of the total splicing product (LE and I) over total proteins (LE+I+P) and total N-cleavage product (NE+NC) over total proteins (NE+NC+P). The 0-h splicing value(s) were subtracted at each time point for baseline correction.

### Kinetic analyses

Since *Mtu* 6×His-tagged WT SufB and SufB mutants (H-5A, H-38A, and SI C1A/N359A) were purified and renatured at different temperatures, after normalizing splicing and cleavage values at different time intervals, the plot was generated by taking the percentage of splicing or cleavage product with respect to time (in min). Next, the curve was fitted in pseudo-first-order kinetics, with an equation Y = Y0 + (Plateau-Y0) * (1-exp(-K*x)) [Where X = time, Y0 = Y value when time (X) = time 0, Plateau = max Y value at time t, K = rate constant, expressed in reciprocal of the x-axis (time units)] in Graphpad Prism software. The fitted curve was generated by automatic outlier elimination fitting in a nonlinear regression equation. The rate constant (K) and *V*_max_ were generated by the software. Half-life (t_1/2_) was calculated by Graphpad Prism using the formula (Ln2/K).

### Western blot

Western blot analysis was performed using an anti-His antibody (Invitrogen, LOT 1902132) to confirm the identity of splicing and cleavage products. Following resolution through SDS/PAGE, test proteins were transferred to a nitrocellulose membrane; at 50 V, 2 h. After a successful transfer, blocking was done with 5% skim milk for 2 h at room temperature. Then the blot was incubated with horseradish peroxidase (HRP)-conjugated anti-His antibody (Invitrogen, LOT 1902132) at 1:5000 dilutions for 16 h at 4^0^C. Then blot was washed with 1× Tris-buffered saline, 0.1% tween 20 (TBST) and developed using enhanced chemiluminescence (ECL) as the substrate. N-extein detection was done with 1:2500 antibody dilution.

### Mass spectrometry and chromatography

After renaturation, proteins were resolved through 4–10% SDS PAGE. Protein identification by mass spectrometry was performed at Central Proteomics Facility, Institute of Life Sciences, Bhubaneswar. In gel digestion of protein and extraction followed the protocol described by Shevchenko et al. [64]. Extracted peptides were desalted by using C18 Zip-Tip (Millipore) and then dried in a centrifugal vacuum concentrator. Peptide samples were reconstituted with 0.1% trifluoroacetic acid (TFA) in 50% acetonitrile (ACN) solution, mixed (1:1) with α-cyano-4-hydroxycinnamic acid solution (C8982; 10 mg/ml) and then spotted on to metal target plate in triplicates. MS and MS/MS spectra were acquired using MALDI TOF/TOF (AbSciex TOF/TOF 5800). Acquired spectra were searched against *Mycobacterium tuberculosis* from NCBInr database using Mascot search algorithm (version 2.0; Matrix Science, Boston, Massachusetts) and protein pilot software (SCIEX, U.S.A.) for protein identification [65,66]. Searches were performed allowing trypsin miscleavage up to 1; Carbamido-methylation of cysteine and oxidation of methionine as variable and fixed modification, respectively. The peptide mass tolerance was set as 100 ppm for precursor ion and 0.8 Da for fragment ion with +1 charge. Since all the splicing and cleavage products were identified as *Mtu* FL-SufB, the acquired spectra were researched against a customized database containing probable splicing products and cleavage products of SufB protein of *Mycobacterium tuberculosis* using mascot search engine in Protein Pilot Software with the same parameter. β-galactosidase (β-gal) and bovine serum albumin (BSA) were run as internal calibration. The protein score, percent coverage, theoretical molecular weight, and iso-electric pH value were obtained. The mass spectrometry proteomics data have been deposited in the ProteomeXchange Consortium via the PRIDE [1] partner repository with the dataset identifier: PXD015199.

Purified and renatured *Mtu* FL-SufB protein was examined by analytical high pressure (or performance) liquid chromatography (HPLC) carried out on an Agilent 1200 series instrument equipped with a Zorbax gf-450 column (6 µm, 9.4 × 250 mm) with a flow rate of 1 ml/min. All HPLC runs used the following solvent: 1:1 water and isopropanol (solvent 1) and 0.1% TFA in water (solvent 2). The column was equilibrated using solvent 1 and solvent 2. A protein sample was inserted into the column and ran for 30 min. The retention time vs protein intensity measured at 280 nm was noted. For reference, we ran 6 µl of Precision plus protein ladder (Bio-Rad 1610374) diluted to 30 µl using sodium phosphate buffer. The retention time vs molecular weight of the known protein standards was measured and plotted to make a standard curve. The unknown protein peaks from the test samples were compared with the standard curve to find out the relative molecular weights. The molecular weights of the expected fragments from the matrix-assisted laser desorption ionization time-of-flight (MALDI-TOF/TOF) MS (Supplementary Table S3) data were compared with the standard curve and molecular weights of the unknown peaks were determined.

### MD simulations

#### Homology modeling of Mtu SufB precursor

A homology model of the *Mtu* FL-SufB precursor was built using the SufB chain of the *E. coli* SufB–SufC–SufD complex (PDB ID: 5AWF, chain A), the *Saccharomyces cerevisiae* intein homing endonuclease (PDB ID: 1VDE, chain A), and the *Thermococcus kodakarensis* homing endonuclease (PDB ID: 2CW7, chain A) using Bioluminate 2.7 from Schrödinger. The model was assessed by Ramachandran plot analysis and PROSA (Z-score) [67].

#### MD simulation analysis

Explicit-solvent MD simulations were performed using GROMACS (V5.1.4) [68] with the OPLS-AA force field [69] and the SPC/E water model [70,71]. The *Mtu* SufB model (846 aa) was solvated in a cubic box with at least 1 nm distance between the protein and the edge of the box, and neutralized with NaCl. The system was minimized with target *F*_max_ of no greater than 1000 kJ mol^−1^ 349 nm^−1^ with steepest descent minimization with a spherical cut-off at 1 nm was imposed on all intermolecular interactions with verlet cut-off scheme [72,73]. The leap-frog algorithm with a timestep of 2 fs [74] and a canonical NVT ensemble was used to run the simulation for 100 ns, with temperature maintained at 300 K through velocity-rescale coupling and no temperature coupling. H-bonds were constrained using lincs with the order of 4 [75]. The particle-mesh Ewald (PME) algorithm was used for implementing long-range electrostatic interactions with the grid dimension of 0.16 nm and interpolation order of 4 [76]. Histidines at positions -5 and -38 were mutated to alanine by homology modeling, and MD simulations were performed on these mutant proteins in an identical fashion. The MD simulation trajectories were analyzed with GROMACS [68] and PyMOL Molecular Graphics System, Version 1.2r3pre, Schrödinger, LLC, and plotted using Origin 8.0.

## Results

### Structural domains of *Mtu* FL-SufB precursor

Pairwise sequence alignment of the *Mtu*-FL-SufB and the intein-less *Msm*-FL-SufB protein sequences using Clustal-W could delineate the intein and extein boundaries for *Mtu*-FL-SufB due to the ∼95% sequence similarity between the extein sequences. The boundaries of the 359-amino acid *Mtu* SufB (*Mtu* Pps1) intein from *M. tuberculosis* strain H37Rv were also affirmed by a Blast search of Inbase. The final demarcation of these structural domains is shown in Supplementary Table S2 and is illustrated in Figure 1A. Multiple sequence alignment with other inteins shows conservation of catalytic cysteines (C1 and C+1), Block-B His67, Block-F Asp77, penultimate His358, and terminal Asn359 (Figure 1B). The presence of these conserved residues suggests that the *Mtu*-FL-SufB precursor (846 aa) is auto-processed by a canonical intein splicing pathway to ligate its N-extein (252 aa) and C-extein (235 aa) to form the native SufB protein (487 aa). The major by-product is the SufB intein (359 aa) containing an intein domain (155 aa) and an endonuclease domain (204 aa), with minor off-pathway products possible due to N-terminal cleavage (594 aa) and C-terminal cleavage (611 aa) [54,77].

### Conservation of His-5 and His-38 in different bacterial species

SufB from different mycobacterial and archaeal species exhibit high sequence similarity (Figure 2A,B). Phylogenetic analysis suggests that these mycobacterial proteins have a common ancestral origin [78]. Cladogram analysis of SufB intein sequences from different mycobacterial species shows significant similarities, except in *Mycobacterium leprae*, *Mycobacterium lepromatosis*, *Mycobacterium triplex* and *Mycobacterium xenopi*, possibly due to different intein insertion sites [17]. In *Ferroplasma*, which belongs to a different kingdom, there is divergence for both the intein [Figure 2C(ii)] and the full precursor [Figure 2C(i)]. These variations in the extein sequences and intein insertion sites suggests some divergent intein evolution and independent intein transfer in different species and kingdoms.

Two conserved histidines were identified in the N-extein region of different mycobacterial and archaeal SufB precursor proteins: His-5 and His-38 (Figure 2A). His-5 is conserved in intein-less SufB proteins from *Staphylococcus aureus*, *Bacillus subtilis*, and certain mycobacteria that use SUF complex as the sole pathway for [Fe–S] cluster generation (Figure 2B) [79,80]. His-38 is conserved in both intein-bearing and intein-less mycobacterial SufB proteins where the SUF system is required to synthesize [Fe–S] clusters (Figure 2B). Identification of two highly conserved metal-chelating residues proximal to N-terminal extein–intein junction raises the possibility of their regulatory roles on cleavage and/or splicing as well as in the functionality of [Fe–S] cluster assembly protein SufB. These two histidines could participate in protein splicing either via direct or indirect interaction with the catalytic residues near cleavage site(s).

### Kinetic study to evaluate the roles of conserved His-5 and His-38 on *Mtu* SufB intein splicing

H-5A and H-38A single mutants were generated to test for a regulatory effect of His-5 and His-38 on *Mtu* SufB intein cleavage and/or splicing. Such alanine substitutions for active site residues have previously resulted in a complete blockage of cleavage and splicing reactions [36]. The SI SufB double mutant, with both C1 and N359 mutated to alanine (C1A/N359A), which is expected to abolish intein splicing completely [81], was used as a negative control.

**Figure 3:**
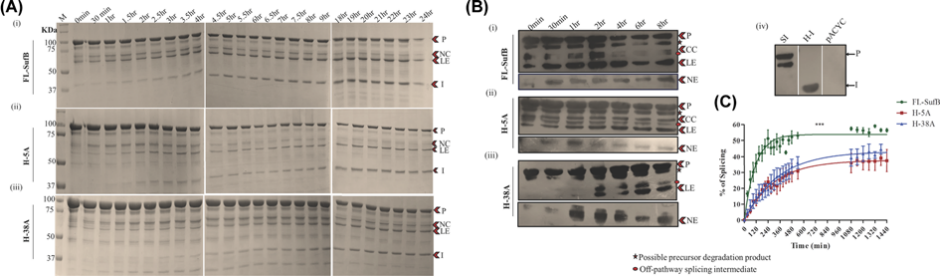
Effect of H-5A and H-38A mutations on *Mtu* SufB splicing (**A**) Products from *in vitro* refolding reactions were resolved through 4–10% gradient SDS PAGE for (i) *Mtu* FL-SufB precursor, (ii) H-5A, and (iii) H-38A mutant proteins exhibiting splicing over different time periods. (**B**) Western blot using anti-His antibodies confirms the identity of splicing and cleavage products for 6×His-tagged (i) *Mtu* FL-SufB precursor,(ii) H-5A, (iii) H-38A, and (iv) the controls SI (splicing inactive double mutant SufB), H-I [6×His-tagged SufB intein], and pACYC Duet-1 (cell lysates expressing empty expression vector). NE was blotted separately with higher concentration of primary antibody. (**C**) Splicing kinetics in *Mtu* FL-SufB precursor, H-5A, and H-38A with product quantities over different time periods fit to a pseudo-first-order reaction; Y = Y0 + (Plateau-Y0) * (1-exp(-K*x)). A statistically significant (***) difference in splicing efficiency (*P*<0.0001) is observed between *Mtu* FL-SufB precursor, H-5A and H-38A mutants. Error bars represents (±1) SEM from three independent sets of experiments. Abbreviations: CC, C-cleavage; I, intein; LE, ligated extein; M, protein marker; NC, N-cleavage; NE, N-extein; P, precursor.

Remnant 6×His-tagged *Mtu* FL-SufB precursor (P, 95.98 KDa), N-terminal cleavage (NC, 65.26 KDa) product, N-extein (NE, 29.9 KDa), ligated exteins (LE, 55.7 KDa), and intein (I, 40.2 KDa) were all observed upon *in vitro* refolding followed by SDS PAGE, and Western blot analysis of the splicing of the *Mtu* FL-SufB precursor and its H-5A and H-38A mutants (Figure 3). As expected, none of the post-reaction products were seen in the case of SI double mutant (Supplementary Figure S2A) and transformants expressing empty vector pACYC Duet-1 (Supplementary Figure S2B). Possibly due to protein degradation, the C-terminal cleavage product (CC, 70.12 kDa) and C-extein (CE, 25.76 kDa) were not detected, which precluded analysis of the effects of H-5A and H-38A mutations on C-terminal cleavage.

#### H-5A and H-38A mutants exhibit attenuated splicing reaction

Densitometric analysis was performed after *in vitro* renaturation of FL and mutant SufB proteins over a period of 24 h. Precursor (P) proteins detected at time 0 h were comparable for FL and mutant proteins (Figure 3A). Splicing efficiency was calculated as a percentage of splicing [(I+LE/P+I+LE) × 100] and plotted over time as pseudo-first-order reaction kinetics (Figure 3C). At 20°C, at least three-fold reductions in splicing efficiency were observed for H-5A and H-38A, relative to *Mtu*-FL-SufB precursor (*P*<0.0001; one-way ANOVA, Table 1).

**Table 1:** Comparative analysis of different kinetic parameters for splicing in FL-SufB precursor, H-5A and H-38A SufB mutant proteins, at 20°C temperature

Kinetic parameters	FL-SufB	H-5A	H-38A
***Y*_max_**	53.81 ± 1.03	37.82 ± 1.68	43.35 ± 1.7
**Rate constant (K) (min^−1^)**	9.92 ± 0.007 × 10^−3^	3.3 ± 0.0003 × 10^−3^	2.9 ± 0.0003 × 10^−3^
**Half-life (t_1/2_)**	1.1 h	3.4 h	3.8 h

### Kinetic studies of *Mtu* SufB N-terminal cleavage

**Figure 4:**
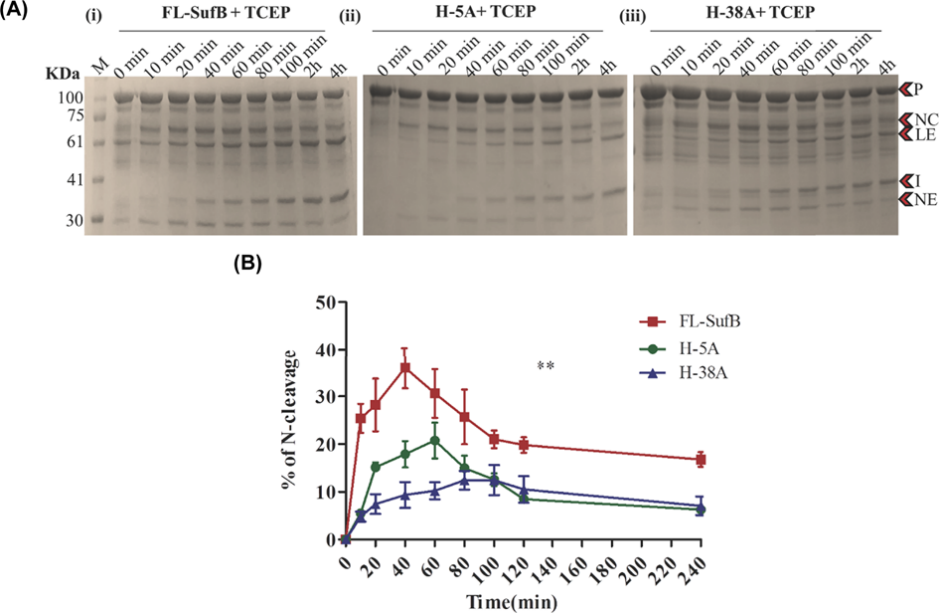
TCEP-mediated N-cleavage in *Mtu* SufB (**A**) SDS PAGE analysis of (i) *Mtu* FL-SufB precursor, (ii) H-5A and (iii) H-38A proteins displaying N-cleavage reactions over different time periods. (**B**) Statistically significant (**) differences (*P*=0.0011, one-way ANOVA) are seen between TCEP-mediated N-cleavage in *Mtu-*FL-SufB (i), H-5A (ii), and H-38A (iii). All the experiments were performed in triplicate and error bars represent (±1) SEM. Abbreviations: CC, C-cleavage; I, intein; LE, ligated extein; NC, N-cleavage; NE, N-extein; P, precursor.

**Figure 5:**
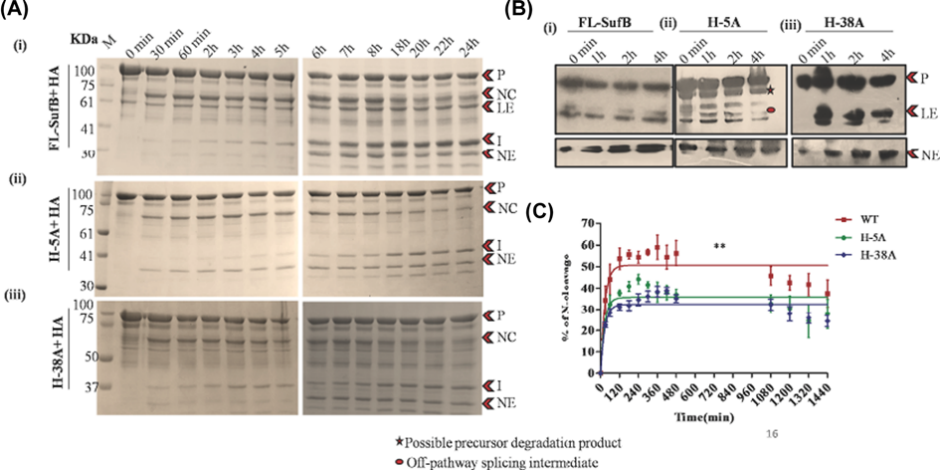
HA-mediated N-cleavage in *Mtu* SufB (**A**) SDS PAGE analysis of HA-induced N-cleavage reactions in (i) *Mtu* FL-SufB precursor, (ii) H-5A, and (iii) H-38A. (**B**) Western blot analysis of splicing and cleavage products for HA-induced N-cleavage reaction in (i) *Mtu* FL-SufB precursor, (ii) H-5A, and (iii) H-38A. NE is blotted separately with higher concentration of primary antibody. (**C**) Kinetic analysis of HA-induced N-cleavage in WT, H-5A and H-38A mutant proteins suggests statistically significant (**) differences (*P*=0.0025; one-way ANOVA) in % of N-cleavage. All the experiments were performed in triplicates and error bars represent (±1) SEM. Abbreviations: CC, C-cleavage product; I, intein; LE, ligated extein; NC, N-cleavage; NE, N-extein; P, precursor..

The equilibrium of the first N/S acyl transfer step was examined using the reagents DTT and Hydroxylamine (HA), which induce N-terminal cleavage reaction. DTT can act both as a nucleophile and reducing agent. HA acts as a nucleophile and like DTT targets the linear thioester intermediate in the first step of splicing [82]. The reducing agent TCEP was used as a control [83]. N-cleavage reaction products were analyzed by SDS PAGE (Figures 4 and 5, and Supplementary Figure S3) and densitometric analysis of the cleavage products.

#### H-5A and H-38A mutations reduce TCEP-mediated N-cleavage

The H-5A and H-38A mutant proteins showed diminished production of N-cleavage (NC) products relative to *Mtu* FL-SufB [Figure 4 and Table 2(i)], although a proper fit for the reaction time course was not obtained through linear or nonlinear regression. In *Mtu*-FL-SufB, N-cleavage was accelerated until 40 min followed by a decline as splicing became distinct. H-5A and H-38A mutants presented a sluggish course with the N-cleavage product peak at 60–80 min and then tapered off. At 40 and 60 min, H-5A displayed 2- and 1.5-fold decrease in % N-cleavage respectively, relative to FL-SufB. Likewise, at 40 and 60 min, H-38A mutant displayed a 3.8- and 3-fold reduction in % of N-cleavage respectively relative to FL-SufB (Figure 4).

**Table 2:** (i) Comparative analysis of TCEP-mediated N-terminal cleavage reaction at 20°C over 240 min; (ii) different kinetic parameters (*Y*_max_, rate constant, and half-life) for HA-induced N-terminal cleavage in FL-SufB precursor, H-5A, and H-38A SufB mutants

(i)			
Time (min)	FL-SufB (% of N-cleavage)	H-5A (% of N-cleavage)	H-38A (% of N-cleavage)
**20**	28.2 ± 5.5	15.1 ± 0.97	7.42 ± 2
**40**	36 ± 4.3	17.89 ± 2.7	9.3 ± 2.7
**60**	30.63 ± 5.1	20.76 ± 3.7	10.2 ± 1.7
**80**	25.7 ± 5.7	14.9 ± 2.5	12.42 ± 1.9
**120**	19.8 ± 1.6	8.4 ± 0.7	10.5 ± 2.7
**240**	16.78 ± 1.5	6.2 ± 0.89	7.07 ± 1.9

(ii)			
Kinetic parameters	FL-SufB	H-5A	H-38A
***Y*_max_**	50.67 ± 1.6	35.72 ± 1.4	32.45 ± 0.94
**Rate constant (K) (min^−1^)**	3.7 ± 0.01 × 10^−2^	3.9 ± 0.01 × 10^−2^	3.7 ± 0.009 × 10^−2^
**Half-life (t_1/2_)**	18.5 min	17.3 min	18.2 min

#### H-5A and H-38A mutations reduce HA-induced N-cleavage

HA is an α-nucleophile that intercepts between amide-ester equilibrium and induces N-cleavage. It enhances the nucleophilicity of residues that leads to N-cleavage [84,85]. A pseudo-first-order fit for the HA-induced reaction time course with the equation, Y = Y0 + (Plateau-Y0) * (1-exp(-K*x)) was obtained (Figure 5). H-5A and H-38A mutants exhibited approximately 1.4- and 1.5-fold reduction (*P*=0.0025; one-way ANOVA) in % of N-cleavage, respectively, relative to *Mtu* FL-SufB although the K and t_1/2_ values were comparable (Figure 5C and Table 2(ii)). This effect on HA-induced N-cleavage reaction was similar to what is observed in presence of TCEP for the H-5A and H-38A mutations.

#### H-5A and H-38A mutations do not alter DTT-induced N-cleavage

Similar to HA, DTT is also a nucleophile that enhances the nucleophilicity of residues and facilitates N- terminal cleavage reaction [83,86]. N-cleavage efficiency was not noticeably different between H-5A and H-38A mutants and *Mtu*-FL-SufB in presence of DTT (Supplementary Figure S3 and Table S4). DTT interacts directly with the residues that may increase the flexibility of the active site structure [87], which possibly counteracts the effect of H-5A and H-38A mutations on N-terminal cleavage. The hypothesized role of the histidines at -5 and -38 positions in the N-extein region in direct or indirect Cys1 activation was probed further by MD simulations.

### Protein identification

The identity of splicing and cleavage products (P, CC, LE, and NE) for the N-terminal 6×His-tagged proteins was affirmed via Western blot using anti-His antibodies (Figures 3B and 5B, and Supplementary Figure S3B). SI (C1A/N359A) double mutant SufB was used as a control that yielded just unspliced precursor protein (P) [Figure 3B(iv)]. One additional protein band was noticed above LE and below CC for *Mtu* FL-SufB, H-5A, and H-38A possibly due to a splicing intermediate as an off-pathway product. We also observed a product just below the P band for *Mtu* FL-SufB, SI, H-5A, and H-38A (Figures 3B and 5B, and Supplementary Figure S3B). However, this protein band was missing in cells expressing SufB intein or empty vector pACYC Duet-1 [Figure 3B(iv)]. This indicated that this band corresponded to a SufB precursor degradation product in the cells overexpressing active and inactive *Mtu* FL-SufB precursor.

To further clarify the identity of these different splicing and cleavage products, MALDI-TOF/TOF mass spectrometry (MS) was performed (Supplementary Table S3) on protein bands cut from the SDS/PAGE gel. When the acquired MS and MS/MS spectra were searched against the taxonomy *Mycobacterium tuberculosis* from NCBInr [88] database, all the splicing products were identified as SufB protein of *Mycobacterium tuberculosis* complex with a statistically significant score (Supplementary Table S3). A further check against a customized database containing probable splicing and cleavage products of SufB protein of *Mycobacterium tuberculosis* with same parameters for the individual protein band spectra resulted in the identification of matching protein splicing products. Individual protein band identifications with protein score, percent coverage, theoretical molecular weight, and isoelectric pH value are shown in Supplementary Table S3. Significance is measured from the expectancy value (with a *P*-value ≤0.05).

Protein fragments of *Mtu* FL-SufB were also found to be within the expected mass range as per retention time (RT) by HPLC (Supplementary Figure S1).

### Proposed mechanism for the cleavage of N-terminal intein–extein peptide bond

**Figure 6:**
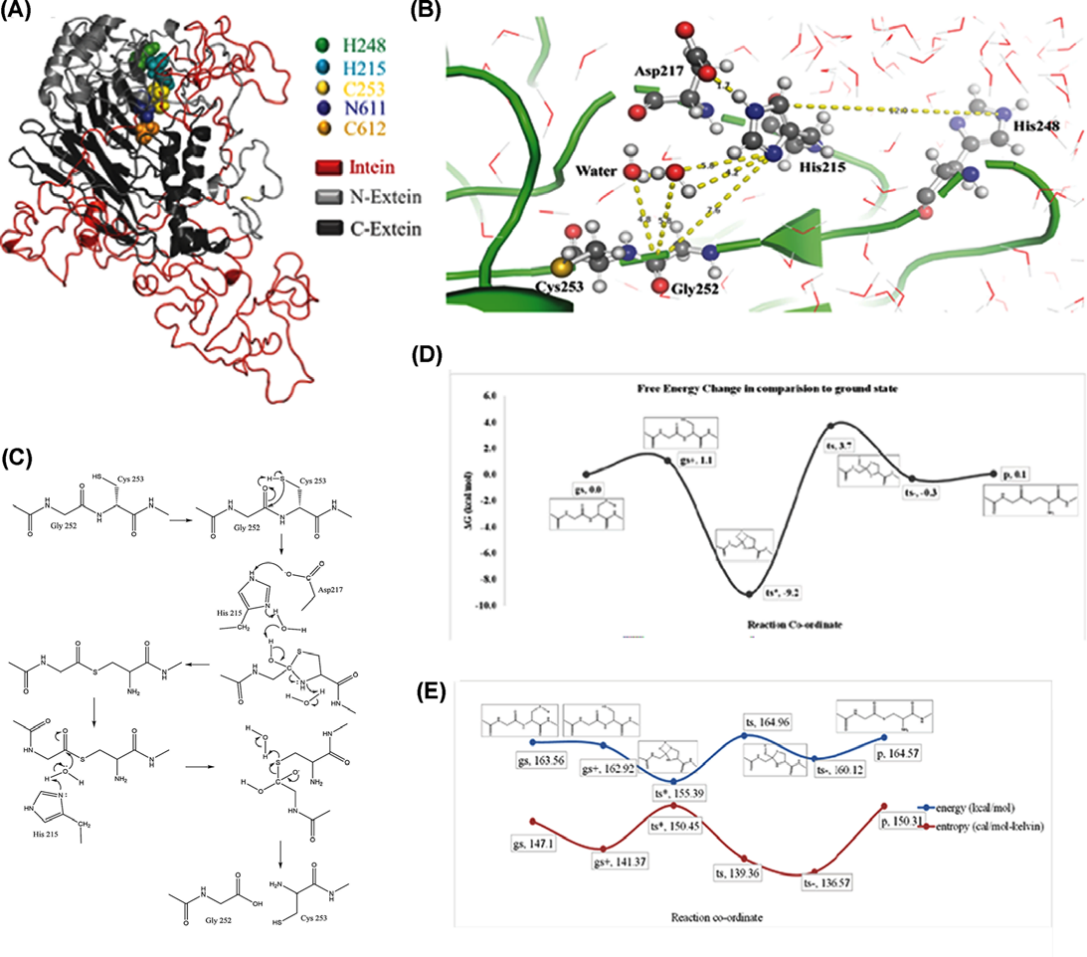
Structural model and proposed mechanism for N-cleavage reaction in *Mtu* FL-SufB precursor (**A**) The 3D model of *Mtu* FL-SufB precursor obtained in explicit solvent. Different domains and conserved catalytic residues are color coded as shown in the legend. (**B**) Spatial arrangement of the N-terminal active site residues; Cys253 (C1) and Gly252 (G-1) along with His248 (H-5), His215 (H-38) and Asp217 (D-36) at the N-terminal cleavage site. (**C**) Proposed mechanism for N-terminal cleavage reaction at intein–extein junction. (**D**) Free energy change, and (**E**) energy and entropy of the system during QM calculation of different reaction states of N-cleavage junction from peptide bond to thioester bond of *Mtu* FL-SufB precursor protein. Abbreviations: gs, ground state; gs+, conformational transition state; p, product; ts, tetrahedral intermediate; ts−, transition state of proton transfer; ts*, transition state of proton transfer.

The *in vitro* experimental results suggest that the mechanism of *Mtu* SufB intein cleavage at the N-terminal cleavage site between Gly252 (G-1) and Cys253 (C1) is influenced by two conserved histidines at -5 (248, full-length protein) and -38 (215, full-length protein) positions in the N-extein sequence of the *Mtu* FL-SufB protein. To further probe the basis of this influence, we performed MD simulations on a three-dimensional (3D) model of the *Mtu* FL-SufB precursor protein (Figure 6A). This also aided identification of possible critical residues at the SufB intein active site (N- and C-terminal splice junctions) (Figure 6B and Supplementary Figure S7), given their proximity to catalytic residues Cys253 (C1), Cys612 (C+1), and Asn611 (N359).

The composite 3D model of *Mtu* FL-SufB precursor was built based on percent sequence identity with analogous sequences from the SufBCD complex of *E. coli* (40% identity, PDB ID: 5AWF, chain A), PI-SceI; a homing endonuclease with protein splicing activity (45% identity, PDB ID: 1VDE, chain A) and intein homing endonuclease II (22% identity, PDB ID: 2CW7, chain A). This model of *Mtu* FL-SufB lacked secondary structure in the intein region due to low sequence identity to the template, with nearly 13% of amino acids are found to be present in the disallowed region in the Ramachandran plot (Supplementary Figure S5A). To optimize this structure further, we carried out MD simulations of the model in explicit solvent for a duration of 100 ns. Based on parameters such as solvent accessible surface area (SASA) (Supplementary Figure S4B) and root mean square deviation (RMSD), the structure was found to be equilibrated after 80 ns (Supplementary Figure S4A). The equilibrated *Mtu* FL-SufB structure was found to have only ∼1% amino acids in the disallowed region of the Ramachandran plot (Supplementary Figure S5B). We found that the longer side chain amino acids in the disallowed region of the Ramachandran plot were mostly solvent-exposed. The ProSAweb score of the model before and after the 100 ns MD simulations is shown in Supplementary Figure S5C,D.

The simulations were also analyzed for the role of specific residues, especially His248 (H-5), which is near the N- and C-cleavage sites. The comparison between 20 ns duration simulations for the *Mtu* FL-SufB precursor and H-5A mutant for RMSD and Root Mean Square Fluctuation (RMSF) parameters is shown in Supplementary Figure S6A,B. It was observed that His248 (H-5) attracts His215 (H-38) towards the active site Gly252 (Gly1), which could facilitate the *Mtu* SufB N-cleavage reaction. Although His-5 is closer to the active site compared with His-38 in the primary sequence, His-38 is closer to the active site in the 3D model. Specifically, the shortest distance between His-38 and Gly1 was observed to be 6.8 Å, which is similar to distances observed between catalytic residues in other inteins [12,39].

Similarly, His610 (His358), Asn611 (His359), and Cys612 (Cys+1) were located near the C-cleavage site in the *Mtu* FL-SufB MD simulations (Supplementary Figure S7). The distance between Asn611 (N359) and Cys612 (C+1) was found to be 5.5 ± 0.1 Å and the distance between Asn611 (N359) and His610 (H358) was 4.3 ± 0.3 Å (Supplementary Table S5). This proximity suggests that the C-cleavage reaction may be facilitated by interactions among these three amino acids, but further analysis of this reaction step is outside the scope of the present study.

Coordination between N-terminal intein–extein splice junction residues Gly252 (Gly1), Cys253 (Cys1), and His215 (His-38), and His248 (His-5) likely propels *Mtu* SufB N-terminal cleavage. Quantum mechanical (QM) calculations were used to obtain energetics of this N-cleavage reaction as done before for intein cleavage of the *Mtu* RecA intein [23]. After the optimized structures of the reactants and the products for each step were obtained, they were used as the initial and final states, respectively. Frequency analyses helped identify the stationary points of the intermediates (only real frequencies) and the saddle points of the transition states (one imaginary frequency). The reaction energy barrier was obtained from the energy difference between the transition state and reactant state with the zero-point correction. A postulated schematic for the cleavage reaction, analogous to the canonical intein splicing mechanism [89], is shown in Figure 6C. All stationary states were confirmed by means of vibrational frequency analysis, and the Gibbs free energy at physiological temperature was calculated for all stationary points, including the zero-point energy, the entropy, and the thermal energy (all at 298.15 K) at the B3LYP/6-31G (d) (Figure 6D,E).

## Discussion

### *Mtu* SufB likely follows a canonical intein splicing mechanism

Being an essential component of the mycobacterial SUF system, the SufB protein is well conserved in mycobacteria, bacteria, and archaea. Intein-carrying SufB proteins are found in different mycobacterial species, although the intein insertion points vary in some species (Figure 2) [54]. Cladogram analysis suggest some divergence in *M. leprae*, *M. lepromatosis*, *M. xenopi*, *M. triplex* and *Ferroplasma* in the extein sequences and intein insertion sites, which suggests intein–extein co-evolution and independent intein transfer in different species and kingdoms. Domain analysis of the *Mtu* FL-SufB precursor (846 aa) clearly demarcates the intein and extein structural regions (Figure 1 and Supplementary Table S2), with the *Mtu* SufB intein (359 residues) containing an intact endonuclease domain. Catalytic residues critical for classic intein splicing pathways like Cys1 (Block A), Cys+1 (Block G), penultimate His (Block G), and terminal Asn (Block G) are conserved in different mycobacterial species including *Mtu*. The *Mtu* SufB intein also contains the conserved Block-B His67 that is known to catalyze the first N–S acyl shift by destabilizing the scissile peptide bond. *In-vitro* refolding of *Mtu* SufB precursor gives splicing and cleavage products that also concur with the roles of these conserved residues. All these observations suggest that *Mtu* FL-SufB undergoes a canonical (classic) *cis*-splicing autoprocessing mechanism to form the functional ligated extein protein (Figure 1).

### A distinct N-terminal cleavage mechanism regulated by conserved H-5 and H-38

Sequence analysis detected two highly conserved His residues in the N-extein sequence of *Mtu* SufB protein; His-38 and His-5 located in all SufB proteins where SUF constitutes the exclusive pathway for [Fe–S] cluster generation irrespective of genus and kingdoms (Figure 2A,B). Catalytic residues such as Cys1, Cys+1, and terminal Asn directly participate in intein splicing by promoting sequential nucleophilic displacement reactions or by rearrangement of bonds near splice junctions. Noncatalytic residues assist indirectly via activation of active site residues and stabilization of various intermediate structure(s). Conserved His residues within intein sequence are known to play important roles during protein splicing as well. Block B His accelerates N–S acyl shift and cleavage of N-terminal intein–extein peptide bond whereas F- and G-Block His are crucial in the coordination of terminal Asn cyclization and cleavage of C-terminal splice site.

Splicing assays and kinetic analysis on *Mtu* FL-SufB precursor and mutant proteins suggests a mechanism where His-5 and His-38 coordinate to facilitate N-cleavage reaction via activation of catalytic Cys1 [Figures 3-5]. Both H-5A and H-38A mutants exhibit a sluggish splicing reaction relative to FL-SufB protein under optimum experimental conditions (Table 1). N-cleavage reaction kinetic analysis shows that H-5A and H-38A mutations reduce N-cleavage efficiency in presence of TCEP and HA (Table 2(i,ii), Figures 4 and 5), but DTT-induced thiolysis is unaffected (Supplementary Figure S3). This may be explained by DTT being a stronger nucleophile that exhibits a more efficient thiolysis and disulfide reduction. H-5 and H-38 therefore seem to assist *Mtu* SufB N-cleavage in conjunction with other active site residues.

To further ascertain the roles of these conserved N-extein histidines, a 3D model of the *Mtu* FL-SufB precursor was built by homology modeling. Explicit solvent MD simulations were performed to optimize and equilibrate the model. H-38 was observed to be localized closer to the active site in the optimized model, which suggests that H-5 and H-38 act in a concerted manner to facilitate *Mtu* SufB N-cleavage. Other active site residues such as Gly1 and Cys1 also seem to be involved. QM/MM calculations using equilibrated structures from the MD simulations provide a quantitative characterization of the energetics of the N-cleavage reaction.

Taken together, these results support the following postulated mechanism for *Mtu* SufB N-cleavage reaction, which is shown as a schematic in Figure 6C. The polarized thiol group of Cys1 approaches the peptidyl C=O, with this transition state being enthalpically favored by an interaction between the thiol H and the peptidyl O atom. The electron density decreases in the C, S, and H atoms while increasing in the O atom. With the imaginary vibrational frequency displacement vector of C=O being towards the thiol, the C and S atoms come closer to form a C–S bond. The proton migration from S to O is highly favored energetically and entropically with a free energy change of −9.2 kcal/mol, which agrees with earlier results [90,91]. The sp2 hybridized peptide carbon becomes sp3 hybridized forming a tetrahedral transition state with a 2-hydroxy thiazolidine ring. This annihilates the peptide resonance to increase the free energy by +3.7 kcal/mol. To regain the sp2 hybridization and resonance stabilization, the 2-hydroxy group expels proton to the environment. In a tetrahedral state, S and N atoms have a Mulliken charge of +0.05 and −0.55. The N atom of the thiazolidine ring accepts a proton from the environment become neutral and forms a thioester intermediate [90,92]. The conversion of a peptide bond into a thioester bond is energetically equivalent at the start as well as the end of the reaction which suggests this process is entropy-driven. The thioester could be hydrolyzed by the imidazole side chain of His-38 mediated by water. Such base catalyzed thioester hydrolysis in an aqueous environment is well known [93]. The least distance between His-38 and His-5 in MD simulations is approximately 6.8 Å suggesting an interaction between these two residues to aid catalysis [12,19].

Asp-36 could exchange a proton with the His-38. The distance fluctuations between His-38 and His-5 in the MD simulations suggest that the solvent-exposed His-5 could attract His-38 towards the N-cleavage site through water-mediated interactions. His-5 could then facilitate thioester hydrolysis by the attacking species (i.e., hydroxide ion) being generated by His-38.

### Biological significance of conserved histidines in the N-extein sequence of *Mtu* SufB

[Fe–S] cluster-bearing proteins have important physiological roles in electron transfer, redox regulation, metabolic pathways, cellular responses to external stimuli, and as regulators of gene expression [45]. The role of the [Fe–S] cluster is closely associated with the functionality of their bound protein framework. In mycobacteria, the SUF system is the sole pathway for [Fe–S] cluster assembly and repair, especially in response to oxidative stress and iron-limiting conditions inside macrophages [46,52]. During such an event, the intracellular Fe supply is either from siderophore chelation or via the metabolism of [Fe–S] clusters associated with specific proteins [45]. Thus, iron homeostasis plays a major role in mycobacterial survival and virulence. SufB is a [Fe–S] cluster scaffold protein and a vital component of the functional SUF system, and indirectly promotes mycobacterial persistence under stress [52]. Further, SufB has been implicated in mycobacterial iron metabolism [45].

Earlier studies have specified the bonding of Fe in [Fe–S] clusters to mostly cysteines from the protein backbone although there is increasing evidence for other ligands such as histidine, aspartate, arginine, threonine, and tyrosine. The most common alternative ligand for [Fe–S] cluster coordination is histidine that is highly conserved with a role in redox tuning and proton-coupled electron transfer [94]. His433(SufB) and His360(SufD) are identified as key protein ligands for the *de novo* [Fe–S] cluster assembly in *E. coli* [51]. It has been shown that these non-cysteine ligands can influence the stability and reactivity of [Fe–S] clusters.

In addition to their possible role in initiating N-cleavage reaction via catalytic Cys1 activation, the conserved His-5 and His-38 residues in the N-extein sequence of *Mtu* SufB may play a role in precursor stabilization. Apart from Cys, His is also an important ligand for metal coordination [51,94], and His-5 and His-38 could coordinate to Fe^+2^/Fe^+3^ during [Fe–S] cluster biogenesis. Confirmation of SufB precursor stabilization by coordination of these His residues to [Fe–S] cluster or to Fe^+2^/ Fe^+3^ would need further studies.

SUF is an exclusive system for biogenesis of [Fe–S] clusters in many pathogenic organisms such as *Staphylococcus aureus*, *Mycobacterium tuberculosis*, *Plasmodium sp.*, and *Toxoplasma*, making it an attractive drug target. For instance, D-Cycloserine is a clinical second-line drug currently used against *M. tuberculosis* and inhibits SufS. SUF is the target system for a polycyclic molecule 882 that has direct interaction with SufC in *S. aureus* [80]. The current work may therefore aid development of novel anti-TB drugs targeting SufB function and stability.

## Supplementary Material

Supplementary Figures S1-S7 and Tables S1-S5

## Data Availability

All the data are available in the manuscript and supplementary data. The accession code for the *Mycobacterium tuberculosis* SufB protein used in the present study is P9WFP7 (UniProtKB/Swiss-Prot) and WP_003407484.1, GI: 397673309 (NCBI protein database). *** Confirmation of the identity of different splicing and cleavage products of Mtu FL-SufB protein by mass spectrometric analysis:*** the data have been deposited in the ProteomeXchange Consortium via PRIDE partner repository with dataset identifier PXD015199. The details of the submission are given below. ** Project Name:** Identification of full length, splicing and cleavage products of SufB protein of SUF-complex of *Mycobacterium tuberculosis*. **Project accession:** PXD015199 **Reviewer account details** *Username:* reviewer73867@ebi.ac.uk *Password:* bRxRcbvP **The homology model for the full-length Mtu SufB protein has been submitted to Model Archive [Project: ma-x807d].** Full-length Mtu SufB protein of SUF complex of *Mycobacterium tuberculosis*; [https://www.modelarchive.org/doi/10.5452/ma-x807d] with the access code: 6pmXRNkvwR.

## Abbreviations

DTT: dithiothreitol
HA: hydroxylamine
HPLC: high pressure (or performance) liquid chromatography
MALDI-TOF/TOF: matrix-assisted laser desorption ionization time-of-flight
MD: molecular dynamics
*Mtu*: *Mycobacterium tuberculosis*
PAGE: polyacrylamide gel electrophoresis
RMSD: root mean square deviation
SASA: solvent accessible surface area
SDS: sodium dodecyl sulfate
SI: splicing inactive
TCEP: Tris (2-carboxyethyl) phosphine
TFA: trifluoro acetic acid
WT: wildtype
